# Is it time for redefining oligometastatic disease? Analysis of lung metastases CT in ten tumor types

**DOI:** 10.1007/s12672-023-00625-2

**Published:** 2023-02-06

**Authors:** Ofer N. Gofrit, Ben Gofrit, Yuval Roditi, Aron Popovtzer, Steve Frank, Jacob Sosna, S. Nahum Goldberg

**Affiliations:** 1 Department of Urology, Hadassah Medical Center, Faculty of Medicine, Hebrew University of Jerusalem, 12000, 91120 Jerusalem, Israel; 2 School of Engineering and Computer Science, Hebrew University of Jerusalem, Jerusalem, Israel; 3 Department of Oncology, Hadassah Medical Center, Faculty of Medicine, Hebrew University of Jerusalem, Jerusalem, Israel; 4 Department of Radiology, Hadassah Medical Center, Faculty of Medicine, Hebrew University of Jerusalem, Jerusalem, Israel

**Keywords:** Oligometastases, Definition, Clonal origin, Cluster of metastases, Prognosis

## Abstract

**Background:**

Oligometastatic disease (OD) is usually defined arbitrarily as a condition in which there are ≤ 5 metastases. Given limited disease, it is expected that patients with OD should have better prognosis compared to other metastatic patients and that they can potentially benefit from metastasis-directed therapy (MDT). In this study, we attempted to redefine OD based upon objective evidence that fulfill these assumptions.

**Methods:**

Chest CTSs of 773 patients with 15,947 lung metastases originating from ten malignancy types were evaluated. The number and largest diameter of each metastasis was recorded. Metastatic cluster was defined as a cluster of two or more metastases with diameter difference ≤ 1 mm. The prognostic power of seven statistical models on overall survival (OS) was analyzed.

**Findings:**

Both the number of metastases and metastatic clusters had a highly significant impact on OS (p < 0.0001, p = 0.003 respectively). Patients with a single metastasis or a single cluster of metastases (regardless of metastases number), equaling 16.2% of all patients, had significantly better prognosis compared to other patients (p = 0.0002). If metastases diameter variability is ignored, as in the standard definition of OD, then patients with 2–5 and 6–10 metastases would have a similar prognosis.

**Interpretation:**

Patients with a single cluster of metastases, theoretically originating from a single clone, have significantly better prognosis compared to patients with more than one cluster. Using this definition can potentially improve the results of MDT. The upper limit of metastases number should be determined by the technical capabilities of the MDT used.

**Supplementary Information:**

The online version contains supplementary material available at 10.1007/s12672-023-00625-2.

## Introduction

Oligometastatic disease (OD) is a theoretical interim condition, with potentially unique biology, placed between localized and full-blown metastatic disease [1, 2]. The rationale for this description is predicated upon the assumption that patients with OD are expected to have better prognosis compared to other patients with metastatic disease and moreover can potentially be cured by metastases directed therapy (MDT). Currently, in most studies, OD is arbitrarily defined as the presence of up to 5 metastatic lesions that can be safely treated with aggressive local therapy [3, 4]. Using this definition, the SABR-COMET open-label trial randomized 99 patients with several malignancy types to treatment with stereotactic ablative radiotherapy (SABR) or to palliative standard-of-care [5]. The treatment arm showed a 22-month benefit in median OS. However, both groups showed the same risk of developing new metastases over time, and only 21.3% of the patients in the treatment arm had > 5 years of survival without recurrence. This suggests that SABR provided good palliation, but in most patients produced no cure [6]. Review of the literature in prostate cancer shows that MDT demonstrates high local control but only a small proportion of the patients remain without progression after 2 years [7]. When the OD concept was employed in renal cell carcinoma, local results were good with 1-year in-field progression free survival of 70%, but only 39% 1-year out of field progression free survival [4]. In CR cancer, OD treatment with SABR provided a 3-year local control of 64.9% but relapse-free survival was only 24.9% [8]. Taken together, these results suggest that many patients currently diagnosed with OD have far more advanced, micrometastatic diseases. Therefore, in many patients, local control is achieved by MDT and perhaps also OS benefit, but cure is rare. Defining OD upon metastases count alone and ignoring size variation, could be responsible for this. It creates an inhomogeneous group of patients with metastases potentially originating from multiple clones implanting at different times. The prognosis of such patients, particularly those with the potential for multiple seeding events is expected to be poor and cure by MDT unlikely. Indeed, the optimal, potentially curable, patient, with OD should have minimal disease originating from a single clone.

In a previous work, it was shown that analysis of metastases diameter variation in chest CTSs enables one to deduce their clonal origin and mode of spread [9]. Here we explored the possibility of using this analysis for refining the definition of the OD to identify which metastatic patients have the greatest chance for cure. Chest CTs, in which metastases diameter is easy to measure, were selected for this task. The prognostic value of seven mathematical parameters of metastases number and diameter was evaluated, which enabled the proposal of a refined, clinically useful definition of OD.

## Methods

### Patients

The data for this study was retrieved from the archives of two referral centers. Patients included in the study were diagnosed in the years 2000–2020 with metastases to the lungs (ICD-9-CM code 197.0) originating in malignancies of the bladder, breast, CR, kidney, melanoma, pancreas, prostate, sarcoma, stomach, and thyroid. The records of these patients and their chest CTSs were studied. Patient’s gender, age, malignancy type, time of cancer and metastases diagnosis, and OS were recorded. The study protocol was approved by the Hadassah Medical Organization (HMO) IRB (# 0650-21-) in accordance with the 1964 Declaration of Helsinki and its later amendments and the local law regarding experiments in humans from 1980 and its amendment from 1999. The committee waived the obligation for informed consent. Exclusion criteria were diagnosis of primary lung cancer or two or more primary malignancy types. Lung metastases largest diameter were manually measured from axial chest CTSs using the electronic calipers.

### Statistical methods

The effect of the following parameters on OS was evaluated using the Kaplan–Meier (Log-Rank) method:

1. Number of metastases.
2. Number of metastatic clusters of (a cluster was defined as two or more metastases with diameter variation of 1 mm or less).
3. Average number of metastases in each cluster.
4. Variability of the metastasis’s diameters.
5. Variability of metastases number between the clusters.
6. Number of single metastases (that do not fit into any cluster).
7. The linear/parallel ratio (LPR) was defined as follows:

$LPR=\frac{\sum \mathrm{c}-\sum \mathrm{i}}{\sum \mathrm{c}+\sum \mathrm{i}}$ where “c” is total number of metastases that can be clustered (with diameter variation ≤ 1 mm) and “i” is the total number of isolated metastases that do not fit into any cluster [9]. LPR was determined by computer code (supplementary material S1).

The total study population included 773 participants with 15,047 metastases (complete dataset available in supplementary material S2). In 51 patients, a single metastasis was found and in 722 patients, two or more metastases (summing to 14,996 metastases) were noted. Analyzing the effect of metastases number on OS was performed using the entire population cohort. The other analyses, that included comparisons of metastases, were done using the population that had two or more metastases. For each parameter studied, multiple potentially clinical useful iterations were run in search of the most discriminative subdivision with respect to OS. Search for confounders was conducted using ANOVA, Fisher’s exact test and t-test as appropriate.

## Results

Patient’s basic parameters and outcome are accessible in supplementary tables S3 and S4. During follow-up, 599 patients (77.5%) died. In 590 patients (98.5%) death was disease specific. In 511 patients (66.1%) metastases to organs other than the lungs were present.

### The effect of number of metastases on OS

**Fig. 1:**
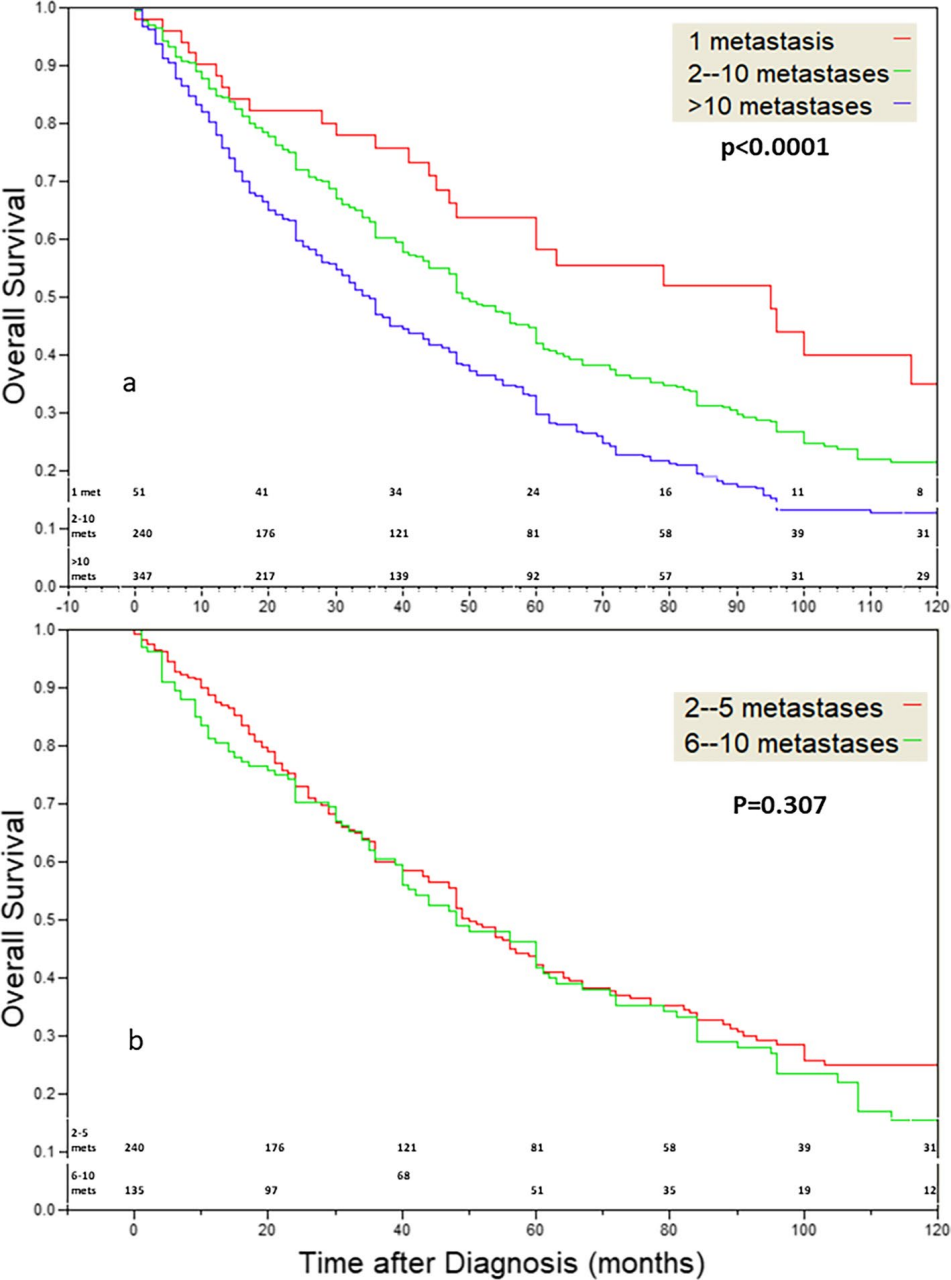
**a** The effect of metastases number on OS. The cutoffs used are: 1, 2–10, > 10 metastases (p < 0.0001). **b** The effect of metastases number on OS. Cutoffs at 2–5 and at 6–10 show an almost identical decay. (p = 0.307)

Patient OS was dependent on the number of metastases (p < 0.0001) when the cutoffs used were: one metastasis, 2–10, and > 10 metastases (Fig. 1a). Patients with a single metastasis had better OS. However, the OS of patients with 2–5 metastases (the standard definition of OD) and 6–10 metastases was similar (Fig. 1b, p = 0.307). Analysis according to tumor type showed that the OS of patients with malignancies of the bladder, CR, kidney, and melanoma was strongly dependent on metastases count, while that of breast, pancreas, prostate, stomach, and thyroid less so (Table 1 and Fig. 1)

**Table 1:** The effect of various metastases measurements on overall survival (Log-Rank, p value)

Primary tumor	Number of metastases^a^	Number of metastatic clusters^b^	Variability of metastases diameters^c^	Average number of metastases in each cluster^d^	Variability of metastases number between clusters^e^	Number of isolated metastasis^f^	Linear/parallel ratio^g^
Bladder	0.004	0.131^h^	0.034	0.069§	0.110	0.068	0.035
Breast	0.1838^h^	0.524^h^	0.570	0.916	0.196	0.557	0.520
Colorectal	< 0.0001	0.032	0.444	0.176	0.002	0.566	0.073
Kidney	0.043	0.027	0.611	0.189^h^	0.066	0.759^h^	0.094
Melanoma	0.024	0.271^h^	0.368	0.662^h^	0.064	0.016^h^	0.140
Pancreas	0.085^h^	0.042	0.316^h^	0.535^h^	0.082	0.093^h^	0.962
Prostate	0.011	0.105^§^	0.342^§^	0.139^h^	0.321	0.482^h^	0.107
Sarcomas	0.110	0.053	0.079	0.426	0.338	0.326	0.593
Stomach§	0.573^h^	0.644^h^	0.175^h^	0.647^h^	0.527	0.326^h^	0.567
Thyroid	0.195^h^	0.0008	0.076^h^	0.054^h^	0.050^h^	0.078	0.031
Overall	< 0.0001	< 0.003	0.078	0.136	< 0.001	0.236	0.002

^a^Comparing: 1, 2–10, > 10 metastases

^b^ Comparing 1–3 clusters to more the 3 clusters of metastases

^c^ Comparing variability in groups: 0–0.1, 0.11–0.2, 0.21–0.3 and 0.31–1

^d^ Comparing averages of 1–2, 2.1–4, 4.1–10, > 10 in a cluster of metastases

^e^ Comparing 0 to > 0

^f^Comparing 0, 1–2, 3–6 and > 6 single metastases

^g^Comparing LPRs of -1–0, 0.1–0.99 and 1

^h^Small number of patients in subgroups

### The effect of number of metastatic clusters on overall-survival

**Fig. 2:**
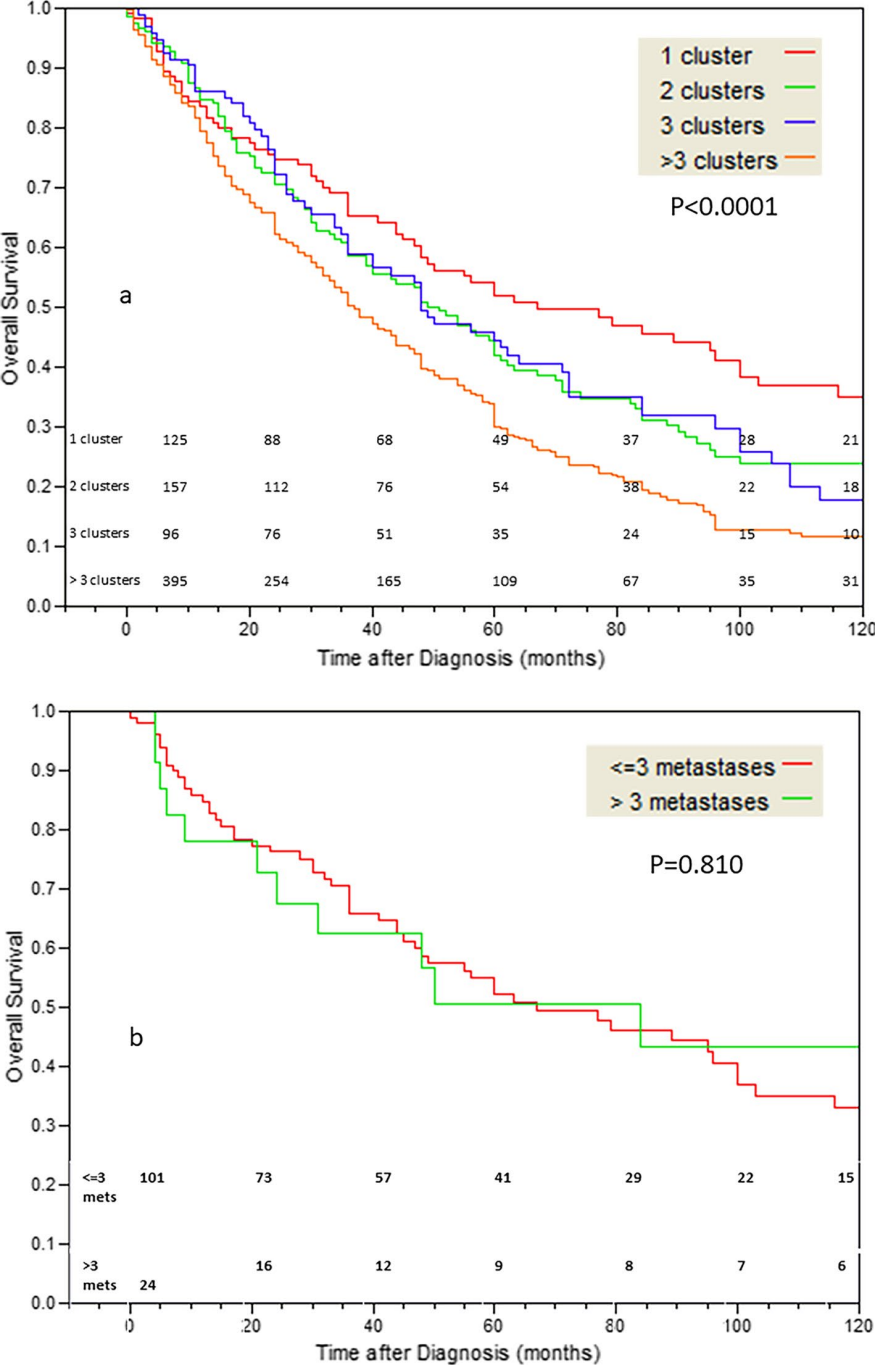
**a** The effect of metastases cluster number (clustering metastases with diameter difference of 1 mm or less) on OS. The cutoffs used are: 1, 2, 3, > 3 clusters (p < 0.0001). **b** The effect of metastases number in patients with a single cluster of metastases comparing patients with <  = 3 metastases to patients with > 3 metastases (4–41 metastases), on OS (p = 0.810)

**Fig. 3:**
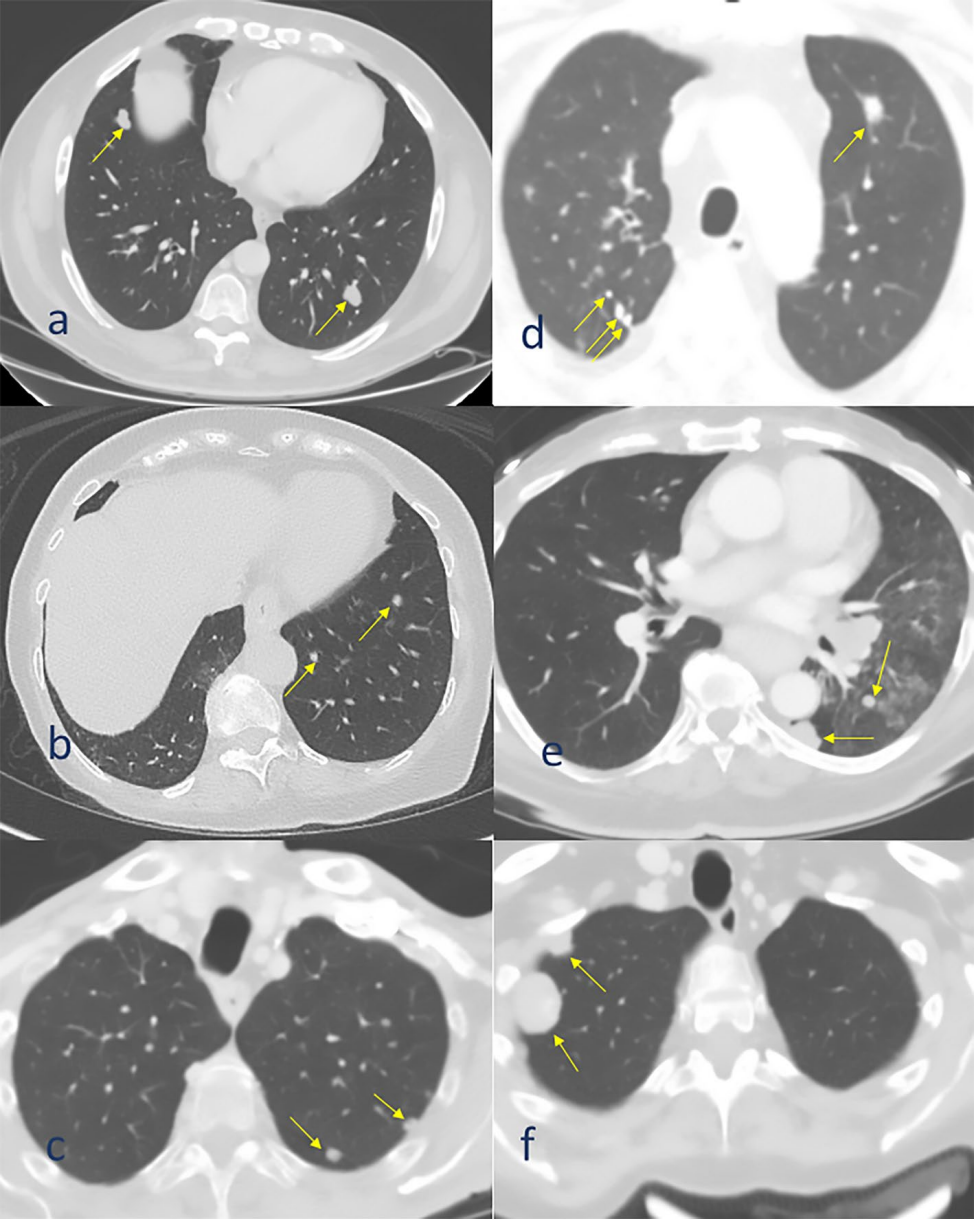
Representative scans of patients diagnosed with a few metastases with similar diameters representing a single (presumably monoclonal spread) cluster. a-liposarcoma, b-pancreas, c-stomach. These patients are suitable for inclusion in an oligometastasis trial. Patients were diagnosed with few metastases but with different diameters (presumably representing multiclonal spread) d-colorectum, e-melanoma, f-osteosarcoma. Despite having only a few metastases, these patients may not be suitable for inclusion in an oligometastasis trial

The number of metastatic clusters had significant impact on OS using the cutoffs: 1, 2, 3 and > 3 clusters (Fig. 2a, p < 0.0001). Patients with a single cluster of metastases (16.2% of all patients), theoretically originating from a single clone, had significantly better OS compared to patients with more than one cluster (p = 0.0002). Malignancies originating in the CR, kidney, pancreas, and thyroid showed significant dependence of OS on cluster number, while those of the bladder, breast, melanoma, prostate, and stomach less so. There were 125 patients with a single cluster of metastases of 1–41 metastases. The OS of patients with a single metastatic cluster of ≤ 3 metastases or > 3 metastases was similar (Fig. 2b, p = 0.810), suggesting that clusters have greater impact on OS compared to number of metastases. Additionally, patients with a single metastasis or a single cluster of metastases showed non-statistically significant differences in OS (p = 0.225), theoretically justifying their grouping together. Examples of patients with a few metastases that represent single or multiple clusters are shown in Fig. 3 (Table 1 and Figs. 2, 3).

The presented data suggests defining the oligometastatic state as a condition in which there is only a single cluster of metastases. In search of confounders to this model, the features of patients with a single and with multiple clusters of metastases were compared (supplementary table S5). Tumor’s origin and patients’ age were similar in patients with single and multiple cluster diseases (p = 0.24 and 0.51 respectively) but there were significantly more men than women in the single cluster group (66.4% Vs. 52.9%, p = 0.06). Sex itself had no effect on overall survival (p = 0.483) and is therefore, not a confounder (supplementary graph S6).

### Analyses of other parameters

Variability of metastases number between clusters and the LPR showed a highly significant impact on OS (p < 0.0001). Per definition, all patients with a single cluster had LPR of + 1. Variability of metastasis diameters, average number of metastases in a cluster and the number of single metastases showed no significant impact on overall survival Table 1.

## Discussion

The concept of OD envisioned by Samuel Hellman in 1995 has attracted much attention in recent years [1–5, 8, 11, 12]. It hypothesizes an interim condition in which a tumor has spread to only a few sites and cure by MDT may be possible. Unfortunately, there is no reliable biomarker for OD and no universally accepted definition [10]. In most studies, OD was arbitrarily defined as a condition in which there are five metastases or less in imaging studies that are technically treatable with MDT [7]. In this study, we suggest adding metastases clonal origin to the definition of OD to identify those patients with greatest chance for cure by MDT.

Tracking a given metastasis’ clonal origin is not an easy task. It requires retrieval of tumorous tissue and complex genetic and epigenetic studies [13–15]. However, in a previous study it was showed that the relatively straightforward analysis of metastases number and diameter may provide readily available information regarding clonal origin by differentiating between linear and parallel patterns of appearance [9]. In brief, the linear model asserts that when a malignant clone gains the capabilities needed to become a metastasis (invasion, angiogenesis etc.); a cluster of metastases disperses from this clone. Since all these metastases have the same clonal origin and commission time, a similar growth rate is expected at any landing site. Thus, all metastases originating in that clone have a similar diameter in any specific organ at any time point. Additional clones may reach this maturity and spread as clusters of metastases but for each cluster, a distinct diameter of all its metastases is expected. By contrast, the parallel model suggests early spread of disseminated tumor cells (DTCs). These cells mature to metastases independently in the target organs and are therefore expected to be at varying diameters. LPR quantitatively displays how much of the tumorous spread was linear and how much parallel. LPR =  + 1 means unmixed linear spread and LPR = − 1 unmixed parallel.

Clearly, the ideal patient to be defined as possessing OD will have a pure a linear (LPR =  + 1), monoclonal, single cluster of metastases, preferably with only a few tumors technically permitting MDT. Patients with a linear multi-clonal or parallel metastatic spread have a more genetically diverse disease and are expected to respond less well to MDT. It is therefore anticipated that a patient with "true" OD will demonstrate a better prognosis compared to patients with non-OD.

In this study using chest CTs of patients with lung metastases originating in ten different malignancies, we showed that patients with a single metastasis or a single cluster of metastases demonstrating diameter variations of 1 mm or less can be classified together into a group possessing significantly better prognosis compared to other patients with metastatic disease (Table 1 and Figs. 1a and 2a). We also showed that if metastases diameter is ignored, the prognosis of patients with 2–5 metastases is similar to that of patients with 6–10 metastases; supporting the concept the five metastases upper limit requirement of OD is probably not biologically true. (Fig. 1b).

Not all tumor types showed the same prognostic dependence on the number of metastases and metastatic clusters. Metastases number had no significant prognostic impact in cancers of the breast, prostate, thyroid, pancreas, and stomach. This is probably due to the small number of patients with a single metastasis in these tumors (only 2, 1, 0, 7, and 3 patients with these tumor types respectively). Metastatic cluster number had no significant impact on the prognosis of patients with tumors of the bladder, breast, melanoma, prostate, and stomach. This could also stem from a small number of patients with a single metastases cluster (only 3, 6, 4, 10, and 8 patients with these tumor types respectively).

Variability of metastases number between clusters and LPR also showed a highly significant impact on OS (p < 0.0001). Both parameters are related to genetic diversity of the metastases. This observation is important scientifically and supports the previous conclusion. However, since these parameters are less intuitive and require computerized calculation, it is suggested not to include them in the definition of the OD. As stated earlier, a single cluster means LPR =  + 1. In search of confounders to this model, the features of patients with single and multiple clusters of metastases were compared (supplementary material S5). Tumor’s origin and patients’ age were similar but there were significantly more men than women in the single cluster group (66.4% Vs. 52.9%, p = 0.06). Gender itself had no significant effect on OS (supplementary material S6) so the significance of this finding is not clear and merits further studies.

The model presented here, with the suggestion of qualifying only patients with a single metastatic cluster (representing a single clone) in the definition of OD, is obviously oversimplified. Yet it could be useful for planning clinical research in OD and may potentially provide better results compared to the poor results obtained with the standard definition of five metastases or less [3–5, 8].

We acknowledge that this study has several notable limitations:

1. Although patients with a single cluster of metastases showed significantly better prognosis, this may not automatically translate into better response to MDT.
2. Metastases growth rate may be influenced by proximity to anatomical structures; thus, metastases originating in a single clone may grow at different rates and mimic several clones.
3. Similarly, an earlier slow growing clone can reach the same diameter as a later but faster growing clone. In this way, several clones may mimic a single clone. Given the improved survival of patients with a single cluster noted according to the proposed model, this condition is unlikely.
4. This study was performed on lung metastases only. Measurement of metastases diameter in other organs is less straightforward and may show different results. Thus, further assessment of these alternative tumor microenvironments will be necessary.
5. Primary tumor control and metastases outside the lungs were not considered in the analysis. As shown by Niibe et al. patients with 1–5 metastases after control of the primary tumor (oligo-recurrent disease) have good prognosis with three-year OS of 64% after SABR [12].
6. Only metastases largest diameter was considered here, due to the simplicity of its measurement. Future studies assessing the potential role of lesion volume and perhaps using deep learning techniques are encouraged.

In summary, this study addresses OD from a biologic/pathophysiologic rather than surgical/anatomic point of view. We demonstrate that patients with a single cluster of metastases, potentially originating from a single clone, even with more than five lesions, have significantly better prognosis compared to patients with polyclonal disease. Accordingly, we propose including monoclonality in the definition of OD. The upper limit of metastases number should not be set arbitrarily and should be determined by the technical capabilities of the MDT. These criteria can potentially improve patient selection for MDT and provide a higher percentage of curative procedures for patients with metastatic disease.

## Supplementary Information

Supplementary: S1. Program code for computation of the LPR.

Supplementary: S2. The complete data set.

Supplementary: S3. Table. Basic parameters of patients with lung metastases.

Supplementary: S4. Table. Outcome of patients with lung metastases.

Supplementary: S5 Table. Comparison of patients with single and multiple clusters.

Supplementary: S6 Graph. a K-M graph showing the effect of sex on overall survival.

## Data Availability

The raw data for this study is provided in supplementary document S2.

## Abbreviations

CR: Colorectum
DTC: Disseminated tumor cells
LPR: Linear parallel ratio
OD: Oligometastatic disease
OS: Overall survival
MDT: Metastasis-directed therapy
SABR: Stereotactic ablative radiotherapy
