# Supplementary material for: Is it time for redefining oligometastatic disease? Analysis of lung metastases CT in ten tumor types
Source: Discov Oncol. 2023 Feb 6;14:19. doi: 10.1007/s12672-023-00625-2 (PMC9902583; doi:10.1007/s12672-023-00625-2)
Supplement: Supplementary file 3 — Supplementary: S3. Table. Basic parameters of patients with lung metastases. [file 12672_2023_625_MOESM3_ESM.docx]

S3: Basic parameters of patients with lung metastases

| Primary  tumour | Number of patients | Average Age at diagnosis (SD) | Gender  F/M (%) | Metastasis present upon diagnosis (%) | Average time from diagnosis to metastases in months (SD)* |
| --- | --- | --- | --- | --- | --- |
| Bladder | 52 | 71.8 (9.0) | 7/45 (13.5) | 18 (34.6) | 31.5 (28.3) |
| Breast | 107 | 56.1 (13.9) | 100/7 (93.5) | 52 (64.2) | 74.8 (59.8) |
| Colorectal | 244 | 60.3 (14.5) | 106/138 (43.4) | 129 (52.9) | 32.6 (26.6) |
| Kidney | 70 | 62.5 (12.6) | 18/52 (25.7) | 26 (37.1) | 52.4 (48.4) |
| Melanoma | 77 | 61.1 (15.0) | 31/46 (40.2) | 28 (36.4) | 39.5 (44.3) |
| Pancreas | 43 | 66.7 (12.3) | 20/23 (46.5) | 40 (93.0) | 26.3 (8.6) |
| Prostate | 34 | 69.9 (10.3) | 0/34 (0) | 23 (67.6) | 58.2 (36.7) |
| Sarcomas | 81 | 51.7 (18.9) | 43/38 (53.1) | 35 (43.2) | 31.0 (45.9) |
| Stomach | 18 | 64.4 (15.9) | 2/16 (12.5) | 13 (72.2) | 11.4 (8.7) |
| Thyroid | 48 | 60.9 (15.3) | 22/26 (45.8) | 25 (52.0) | 66.1 (70) |
| Overall | 773 | 60.8 (15.4) | 347/4426 (44.9) | 387 (50.0) | 43.5 (49.1) |

*In patients presenting without metastases
