# Supplementary material for: Is it time for redefining oligometastatic disease? Analysis of lung metastases CT in ten tumor types
Source: Discov Oncol. 2023 Feb 6;14:19. doi: 10.1007/s12672-023-00625-2 (PMC9902583; doi:10.1007/s12672-023-00625-2)
Supplement: Supplementary file 4 — Supplementary: S4. Table. Outcome of patients with lung metastases. [file 12672_2023_625_MOESM4_ESM.docx]

**S4. Outcome of patients with lung metastases**

| Primary tumour | Average follow-up in months (SD)* | Died (Disease specific) | 2-year overall survival |
| --- | --- | --- | --- |
| Bladder | 33.4 (32.7) | 83.0% (100%) | 52.7% |
| Breast | 73.3  (64.1) | 84.1% (100%) | 77.0% |
| Colorectal | 47.3 (32.9) | 74.2% (98.9%) | 78.2% |
| Kidney | 63.5 (73.3) | 80% (96.4%) | 55.9% |
| Melanoma | 46.9 (46.2) | 74.0% (94.9%) | 60.1% |
| Pancreas | 13.8 (13.2) | 88.6% (100%) | 21.6% |
| Prostate | 66.1 (48.8) | 76.5% (96.2%) | 84.9% |
| Sarcomas | 41.2 (46.2) | 71.6% (100%) | 62.6% |
| Stomach | 12.2 (10.5) | 94.5% (100%) | 12.2% |
| Thyroid | 90.4 (90.9) | 62.5% (96.6%) | 78.9% |
| Overall | 51.9 (53.8) | 77.3% (98.5%) | 67.4% |

*From diagnosis to censoring
