# Supplementary material for: Is it time for redefining oligometastatic disease? Analysis of lung metastases CT in ten tumor types
Source: Discov Oncol. 2023 Feb 6;14:19. doi: 10.1007/s12672-023-00625-2 (PMC9902583; doi:10.1007/s12672-023-00625-2)
Supplement: Supplementary file 5 — Supplementary: S5 Table. Comparison of patients with single and multiple clusters. [file 12672_2023_625_MOESM5_ESM.docx]

Table: Comparison of patients with single and multiple clusters

| Feature | Patients with Single cluster | Patients with multiple clusters | P Value |
| --- | --- | --- | --- |
| Origin  Bladder  Breast  Colo-rectum  Kidney  Melanoma  Pancreas  Prostate  Sarcomas  Stomach  Thyroid  Total | 4 patients  8 patients  36 patients  18 patients  16 patients  12 patients  11 patients  4 patients  11 patients  5 patients  125 patients | 48 patients  99 patients  208 patients  52 patients  61 patients  31 patients  23 patients  76 patients  7 patients  43 patients  648 patients | 0.24 |
| Average Age (SD) | 61.6 (14.4) | 60.7 (15.6) | 0.51 |
| Female/Male | 42/83 (33.6%/66.4%) | 305/343 (47.1%/52.9%) | 0.006 |
