# Supplementary figures and images for: Is it time for redefining oligometastatic disease? Analysis of lung metastases CT in ten tumor types
Source: Discov Oncol. 2023 Feb 6;14:19. doi: 10.1007/s12672-023-00625-2 (PMC9902583; doi:10.1007/s12672-023-00625-2)

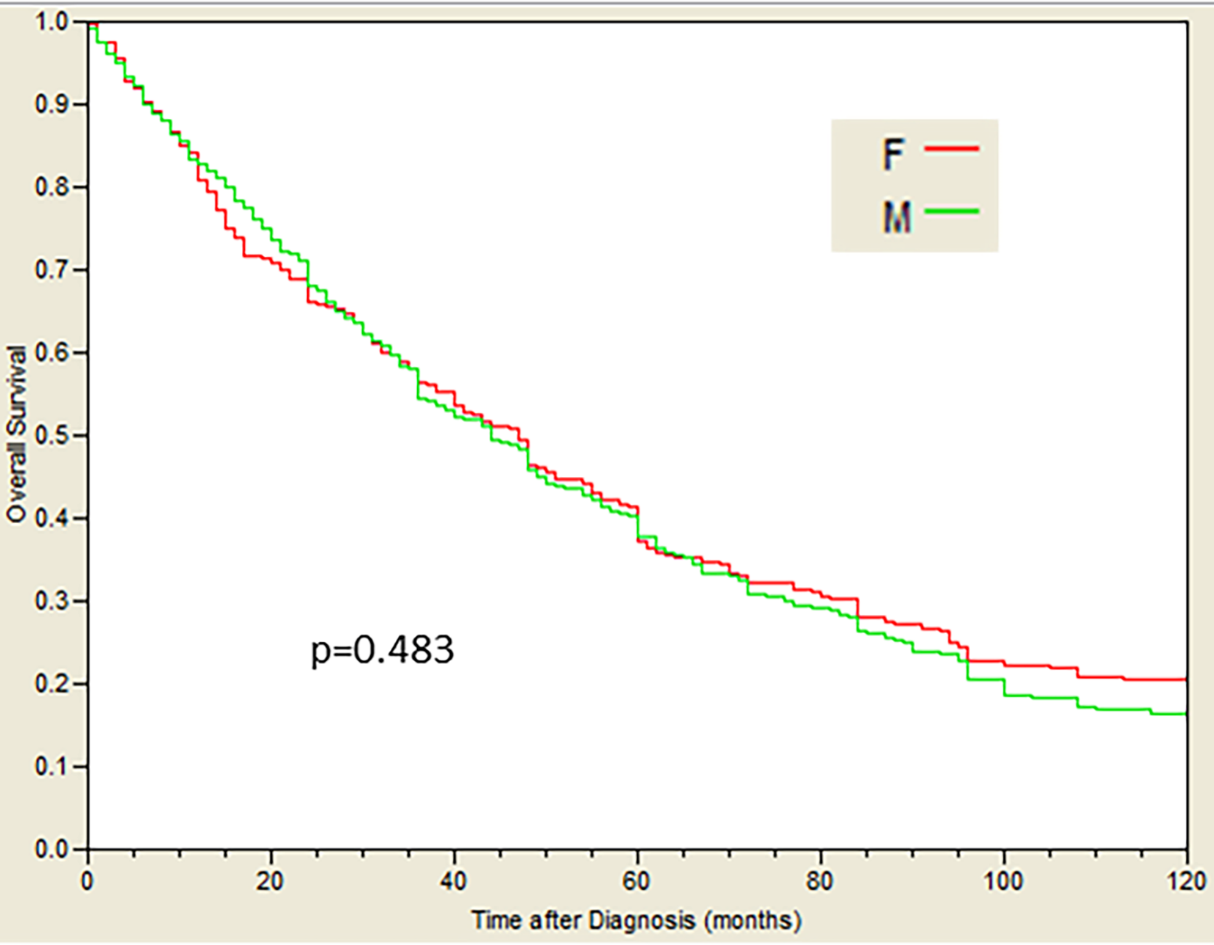


Legend: K-M graph showing the effect of sex on overall survival

Supplement: Supplementary file 6 — Supplementary: S6 Graph. a K-M graph showing the effect of sex on overall survival. [file 12672_2023_625_MOESM6_ESM.docx]
